# The association between triglyceride-glucose index and neutrophil-lymphocyte ratio and all-cause mortality in the general US population: NHANES 2001–2018

**DOI:** 10.3389/fendo.2024.1513543

**Published:** 2024-12-10

**Authors:** Yifei Wang, Cheng Gu, Bingbing Chen, Binxu Qiu, Jinhai Yu

**Affiliations:** ^1^ Department of Gastric and Colorectal Surgery, General Surgery Center, The First Hospital of Jilin University, Changchun, China; ^2^ Department of Joint Surgery, The First Affiliated Hospital, Sun Yat-sen University, Guangzhou, China; ^3^ Department of Laboratory Medicine, Med+X Center for Manufacturing, Department of General Surgery, National Clinical Research Center for Geriatrics, West China Hospital, Sichuan University, Chengdu, China; ^4^ Breast Center, West China Hospital, Sichuan University, Chengdu, China

**Keywords:** TyG, NLR, insulin resistance, mortality, NHANES

## Abstract

**Background:**

The association between the triglyceride-glucose (TyG) index and mortality in the general population remains controversial, with inconsistent findings across studies.

**Objective:**

This study aims to investigate the relationship between the TyG index and mortality in the U.S. Additionally, it explores whether a new index, combining the TyG index with the neutrophil-to-lymphocyte ratio (NLR), improves the prediction of all-cause compared to the TyG index alone.

**Methods:**

Systemic inflammatory markers and the TyG index were calculated based on participants’ complete blood counts and fasting triglyceride and glucose levels. The TyG-NLR index was derived by multiplying the TyG index by the NLR. A weighted Cox proportional hazards model was used to evaluate the associations of the TyG and TyG-NLR indices with mortality risk in the general population. Restricted cubic splines (RCS) were applied to explore and visualize the dose-response relationships between the indices and mortality.

**Result:**

This study included 15388 participants. During a median follow-up of 118 months, 2,333 participants died. After adjusting for potential confounders, no significant association was found between the TyG index and mortality. However, compared to the lowest quartile, participants in the highest quartile of the TyG-NLR index showed a significant association with all-cause mortality. Specifically, those in the highest quartile had a 63% higher risk of all-cause mortality.

## Introduction

Insulin resistance (IR) is a key feature of metabolic syndrome, characterized by a reduced ability of insulin to promote glucose uptake (1–3). Although the hyperinsulinemic-euglycemic clamp test is the gold standard for measuring IR, it is invasive and unsuitable for clinical studies (4, 5). The triglyceride-glucose (TyG) index, calculated from fasting triglyceride and glucose levels, has been shown to be a valuable alternative indicator of IR (6). Due to its simplicity, accessibility, and low cost, the TyG index is widely used in clinical settings. Several studies have investigated the association between the TyG index and mortality in individuals with metabolic disorders, highlighting its effectiveness in predicting adverse outcomes (7–9). However, factors such as sex, age, race, comorbidities, and income appear to influence the association between the TyG index and all-cause mortality in the general population, leading to inconsistent findings across studies.

To enhance the utility of the index, we took into account the combined impact of IR and systemic inflammation, as these factors are known to synergistically drive the progression of metabolic disorders and the onset of adverse health outcomes (10). In this context, the neutrophil-to-lymphocyte ratio (NLR) has emerged as a vital biomarker for evaluating systemic inflammation. Numerous studies have highlighted the significance of NLR in assessing the severity of inflammatory responses, making it an essential parameter for clinical evaluations (11–13). By addressing the interplay between IR and inflammation, we aimed to improve the predictive power of existing tools. This approach not only underscores the interconnected nature of metabolic and inflammatory pathways but also highlights the importance of utilizing comprehensive biomarkers that reflect multiple physiological processes.

Thus, we developed the TyG-NLR index by combining the TyG index with NLR and evaluated its association with all-cause mortality risk in the general population. This longitudinal cohort study aims to determine whether the TyG-NLR index is more effective than the TyG index alone in predicting all-cause mortality risk in the U.S. general population.

## Methods

### Data sources and outcome definitions

The National Health and Nutrition Examination Survey (NHANES) is a research program led by the U.S. Centers for Disease Control and Prevention (CDC) to assess the health status of adults and children nationwide. Initiated in 1960, it has operated as a continuous program since 1999, surveying approximately 5,000 individuals annually. The survey collects comprehensive data on demographics, socioeconomic status, diet, and health (14, 15). This study utilized data from 10 NHANES cycles spanning 1999 to 2018. Additionally, NHANES data were linked with the National Death Index (NDI) to obtain follow-up information, including follow-up duration, survival status, and causes of death (16).

### The TyG index and TyG-NLR index

The TyG index is calculated by taking the natural logarithm of the product of fasting triglyceride and glucose levels for each participant. Systemic inflammation indicators are derived from complete blood counts. TyG-NLR is obtained by multiplying the TyG index by various systemic inflammation indicators (17). The formulas for these calculations are as follows:


TyG=In[triglyceride (mg/dL)×fasting blood glucose (mg/dL)/2]

TyG−NLR=TyG×NLR

### Covariates

The NHANES dataset provided all variables used in this study, including gender, age, race, education level, and socioeconomic status. Other variables included smoking status, alcohol consumption, medical history, medication use, body mass index (BMI), complete blood counts, and biochemical blood tests. Dyslipidemia was defined using multiple parameters: low-density lipoprotein cholesterol (LDL-C) ≥ 3.37 mmol/L, total cholesterol (TC) ≥5.18 mmol/L, triglycerides (TG) ≥ 1.7 mmol/L, and high-density lipoprotein cholesterol (HDL-C) <1.04 mmol/L for men or < 1.30 mmol/L for women (18). The use of lipid-lowering medications was also considered in diagnosing dyslipidemia. Hypertension was defined by an average systolic blood pressure ≥ 140 mmHg and/or diastolic blood pressure ≥ 90 mmHg (19). Diabetes was diagnosed based on physician confirmation, fasting blood glucose ≥ 7.0 mmol/L, HbA1c ≥ 6.5%, or current use of antidiabetic medications (20). Smoking status was classified into never smokers (fewer than 100 cigarettes in a lifetime), former smokers (previously smoked but quit), and current smokers (≥ 100 cigarettes in a lifetime and currently smoking) (21). Alcohol consumption was categorized as heavy drinking (≥ 3 drinks/day for women or ≥ 4 drinks/day for men, or ≥ 5 days/month), moderate drinking (≤ 2 drinks/day for women or ≤ 3 drinks/day for men, or ≥ 2 days/month), light drinking (below moderate or heavy drinking levels), and non-drinkers (lifetime consumption < 12 drinks) (22). Medication use and cancer history were determined via survey responses (23).

### Statistical analysis

All analyses were performed using R software (version 4.2.2), with statistical significance set at *P* < 0.05. Categorical variables are presented as frequencies and percentages, while continuous variables are reported as medians with interquartile ranges (IQR). The Kaplan-Meier method was used to estimate survival probabilities with the log-rank test. We employed three models to assess the impact of potential confounders. Model 1 was unadjusted, Model 2 adjusted for sex and age, and Model 3 further adjusted for race, PIR, education, BMI, smoking status, alcohol consumption, hypertension, diabetes, dyslipidemia, cancer, lipid-lowering medications, ALT, and AST. A restricted cubic spline (RCS) with four knots was used to explore the dose-response relationship between TyG-NLR and mortality.

## Results

### Participant characteristics

**Figure 1:**
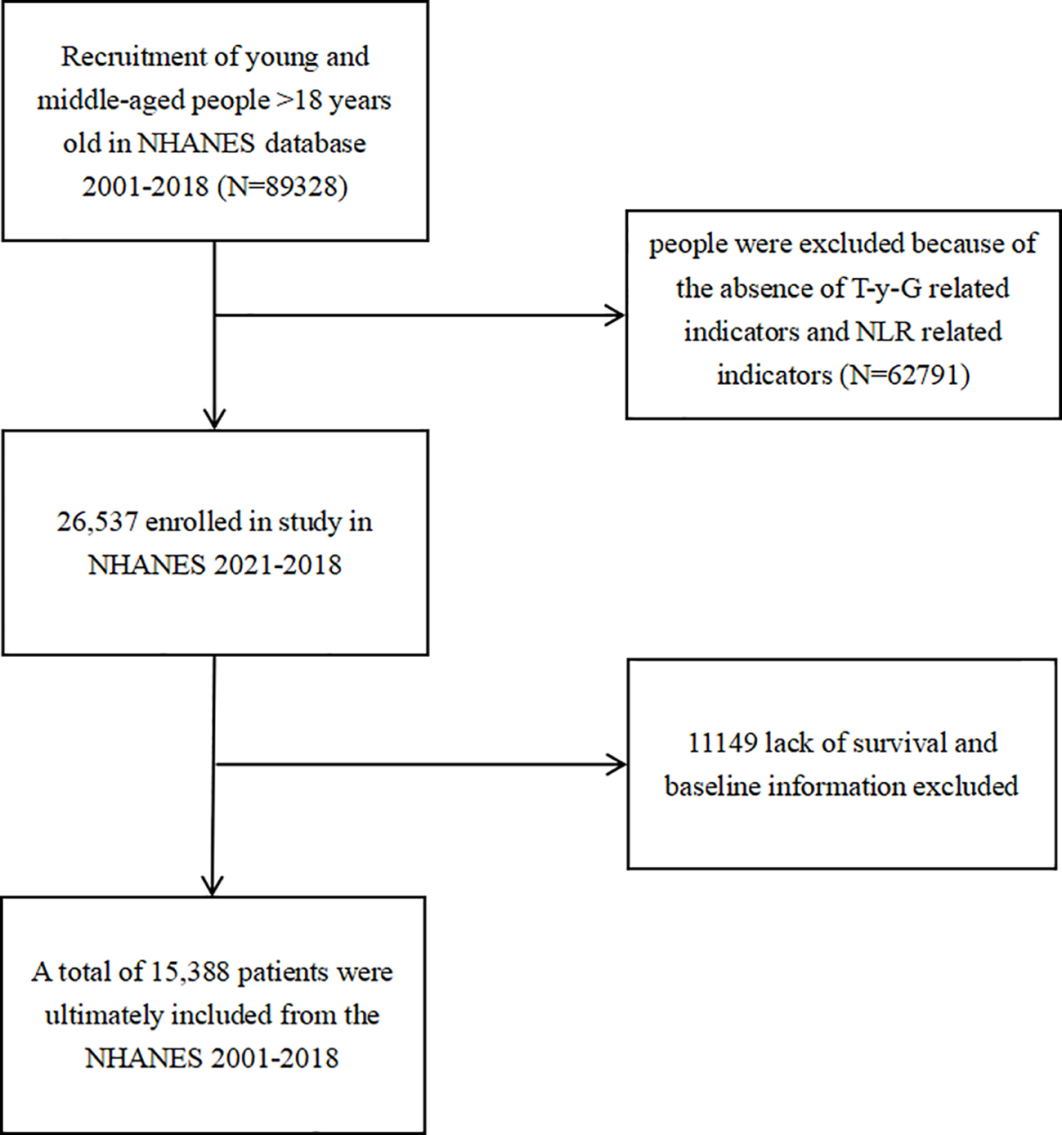
The flowchart of the screening of the selected population.

This study included a total of 15,388 participants, with 52.48% being female (**Figure 1**). During a median follow-up period of 118months, 2,333 participants experienced outcome events. **Table 1** outlines the baseline characteristics of participants in the survivor and non-survivor groups, highlighting significant demographic, clinical, and biochemical differences. Compared to survivors, non-survivors were significantly older, with 69.19% being 60 years or older, compared to 22.35% in the survivor group. They were more likely to be male (38.36% vs. 49.15%) and predominantly non-Hispanic White (63.20% vs. 61.08%). Socioeconomic disparities were evident, with non-survivors having a higher proportion of individuals with a PIR < 1 (13.84% vs. 7.65%) and lower education levels, with only 17.66% having achieved more than high school education compared to 60.99% among survivors. Clinically, non-survivors exhibited a higher burden of comorbidities, including hypertension (93.78% vs. 69.64%), hyperlipidemia (39.15% vs. 30.94%), diabetes, and cancer (37.96% vs. 16.43%). Additionally, a higher proportion of non-survivors were on lipid-lowering medications (19.48% vs. 18.37%). These findings suggest a higher prevalence of chronic conditions and associated cardiovascular risks among non-survivors. Biochemically, non-survivors exhibited higher levels of fasting blood glucose (5.87 vs. 5.40 mmol/L), triglycerides (1.35 vs. 1.08 mmol/L), LDL-C (2.90 vs. 2.58 mmol/L), and aspartate aminotransferase (21.00 vs. 19.00 U/L). They also displayed elevated markers of inflammation, with higher levels of white blood cells (6.70 vs. 6.20 10^9/L), neutrophils (4.00 vs. 3.30 10^9/L), and monocytes (0.60 vs. 0.40 10^9/L), alongside lower lymphocyte levels (1.70 vs. 1.90 10^9/L), indicating a heightened inflammatory state and potential immune suppression.

**Table 1:** Characteristics of participants.

Variables	Total (n = 15388)	Survivors (n = 13055)	Non-survivors (n =2333)	*P-value*
Age group, n (%)	15388	13055	2333	*<0.001*
60	10856 (70.55)	10137 (77.65)	719 (30.82)	
≥ 60	4532 (29.45)	2918 (22.35)	1614 (69.18)	
Sex, n (%)				*<0.001*
Female	8076 (52.48)	6638 (50.85)	1438 (61.64)	
Male	7312 (47.52)	6417 (49.15)	895 (38.36)	
PIR, n (%)				*<0.001*
1	1303 (8.47)	980 (7.51)	323 (13.84)	
1–3	4593 (29.85)	3997 (30.62)	596 (25.55)	
3	9492 (61.68)	8078 (61.88)	1414 (60.61)	
Race, n (%)				*<0.001*
Non-Hispanic Black	1971 (12.81)	1475 (11.30)	496 (21.26)	
Mexican American	1514 (9.84)	854 (6.54)	660 (28.29)	
Non-Hispanic White	11084 (72.03)	10464 (80.15)	620 (26.58)	
Other Race	819 (5.32)	262 (2.01)	557 (23.87)	
Education levels, n (%)				*<0.001*
high school	1976 (12.84)	675 (5.17)	1301 (55.77)	
= high school	5038 (32.74)	4418 (33.84)	620 (26.58)	
high school	8374 (54.42)	7962 (60.99)	412 (17.66)	
Smoking status, n (%)				*<0.001*
Never	7752 (50.38)	6941 (53.17)	811 (34.76)	
Former	3674 (23.88)	2979 (22.82)	695 (29.79)	
Current	3962 (25.75)	3135 (24.01)	827 (35.45)	
Alcoholconsumption, n (%)				*<0.001*
Never	1733 (11.26)	1482 (11.35)	251 (10.76)	
Former	2285 (14.85)	1222 (9.36)	1063 (45.56)	
Mild	6207 (39.17)	5376 (41.18)	831 (35.62)	
Moderate	2341 (15.21)	2249 (17.23)	92 (3.94)	
Heavy	3002 (19.51)	2726 (20.88)	276 (11.83)	
Diabetes, n (%)				*<0.001*
No	10809 (70.24)	9380 (71.85)	1429 (61.25)	
IFG	1328 (8.63)	1074 (8.23)	254 (10.89)	
IGT	1470 (9.55)	1116 (8.55)	354 (15.17)	
Yes	1781 (11.57)	1485 (11.37)	296 (12.69)	
Hyperlipidemia, n (%)				*<0.001*
No	9364 (60.85)	9089 (69.16)	275 (11.79)	
Yes	6024 (39.15)	3966 (30.84)	2058 (88.21)	
Hypertension, n (%)				*<0.001*
No	13640 (88.64)	11452 (87.72)	2188 (93.78)	
Yes	1748 (11.36)	1603 (13.28)	145 (6.22)	
Cancer, n (%)				*<0.001*
No	4850 (31.52)	2798 (16.43)	2052 (87.96)	
Yes	10538 (68.48)	10257 (78.57)	281 (12.05)	
Lipiddrug, n (%)				*<0.001*
No	12521 (81.37)	10512 (80.52)	2009 (86.11)	
Yes	2867 (18.63)	2543 (19.48)	324 (13.89)	
Laboratory data				
FBG (mmol/L)	5.40 (5.07,5.85)	5.30 (5.05,5.70)	5.87 (5.44,6.37)	*<0.001*
TG (mmol/L)	1.10 (0.85,1.70)	1.08 (0.84,1.63)	1.35 (1.03,1.87)	*<0.001*
LDL-C (mmol/L)	2.85 (2.30,3.45)	2.80 (2.34,3.36)	2.90 (2.35,3.67)	*<0.001*
ALT (U/L)	20.00 (15.00,27.00)	19.00 (16.00,25.00)	21.00 (17.00,25.00)	*<0.001*
AST (U/L)	22.00 (18.00,27.00)	22.00 (19.00,27.00)	23.00 (20.00,27.00)	*<0.001*
WBC (10^9^/L)	6.20 (5.10,7.60)	6.00 (5.20,7.60)	6.70 (5.50,8.00)	*<0.001*
PLT (10^9^/L)	240.00 (200.00,285.00)	235.00 (200.00,268.00)	250.00 (210.00,280.00)	*<0.001*
Neutrophils (10^9^/L)	3.40 (2.70,4.40)	3.30 (2.60,4.40)	4.00 (3.00,4.90)	*<0.001*
Monocytes (10^9^/L)	0.50 (0.40,0.60)	0.50 (0.40,0.60)	0.60 (0.40,0.70)	*<0.001*
Lymphocytes (10^9^/L)	1.80 (1.50,2.20)	1.90 (1.60,2.30)	1.70 (1.30,2.20)	*<0.001*
Follow-up time (month)	118 (85,182)	127 (75,173)	106 (65,156)	*<0.001*

BMI, body mass index; PIR, poverty income ratio; IFG,impaired fasting glucose; IGT, impaired glucose tolerance; FBG, fasting blood glucose; TG, triglyceride; LDL-C, low-density lipoprotein cholesterol; ALT, alanine aminotransferase; AST, aspartate aminotransferase; WBC, white blood cell; PLT, platelet; TyG, triglyceride-glucose; NLR, neutrophil-lymphocyte ratio.

### Kaplan-Meier survival curves and the association between TyG, TyG-NLR, and all-cause mortality

**Figure 2:**
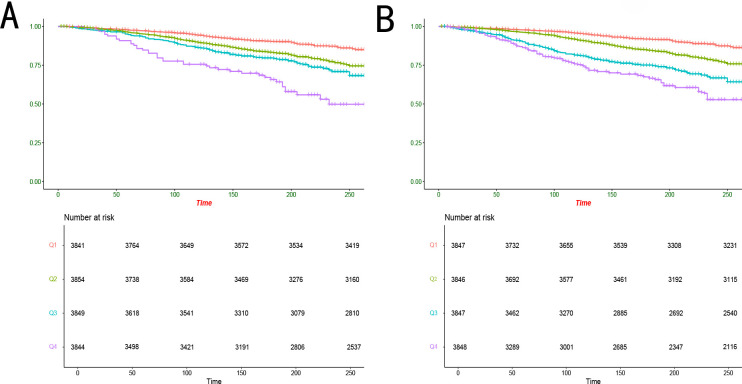
Kaplan-Meier analysis of all-cause mortality with different quartile levels of TyG and TyG-NLR indices. TyG, triglyceride-glucose; NLR, neutrophil-lymphocyte ratio.

Kaplan-Meier survival analysis adjusted for all covariates based on Model 3 showed significant differences in long-term survival outcomes across groups with different TyG index levels (**Figure 2A**, *P*< 0.001). A similar pattern was observed for the TyG-NLR index (**Figure 2B**, *P*< 0.001). We employed three models to evaluate the association between the TyG and TyG-NLR indices and all-cause mortality (**Table 2**, **Table 3**). In Model 3, no significant association was observed between the TyG index and all-cause mortality across the quartiles, with hazard ratios (HRs) ranging from 0.79 (95% confidence interval [CI]: 0.51–1.15) in the third quartile to 1.02 (95% CI: 0.62–1.48) in the highest quartile (**Table 4**). In contrast, the TyG-NLR index demonstrated a significant positive association with all-cause mortality across all models. In Model 3, participants in the highest quartile of the TyG-NLR index had a significantly higher risk of all-cause mortality compared to those in the lowest quartile (HR = 1.63, 95% CI: 1.08–2.54). These findings suggest that while the TyG index alone may not robustly predict mortality risk, the combined TyG-NLR index effectively captures the synergistic impact of insulin resistance and systemic inflammation, making it a more reliable predictor of all-cause mortality.

**Table 2:** Baseline characteristics of the study population based on TyG quartile grouping.

Variables	Total (N=15388)	Q1	Q2	Q3	Q4	*P*-value
Age group, n (%)	15388	3841	3854	3849	3844	*< 0.001*
< 60	10856 (70.55)	3380 (83.95)	2842 (75.40)	2592 (71.35)	2433 (67.46)	
≥ 60	4532 (29.45)	893 (16.05)	1445 (24.60)	1690 (28.65)	1843 (32.54)	
Sex, n (%)						*<0.001*
Female	8076 (52.48)	2542 (61.05)	2138 (50.97)	2015 (46.85)	1863 (43.13)	
Male	7312 (47.52)	1731 (38.95)	2149 (49.03)	2267 (53.15)	2413 (56.87)	
Race, n (%)						*<0.001*
Non-Hispanic Black	1971 (12.81)	588 (15.31)	508 (13.18)	472 (12.26)	403 (10.48)	
Mexican American	1514 (9.84)	268 (6.98)	341 (8.85)	397 (10.31)	508 (13.22)	
Non-Hispanic White	11084 (72.03)	2607 (67.87)	2757 (71.54)	2859 (74.27)	2861 (74.43)	
Other Race	819 (5.32)	378 (9.84)	248 (6.43)	121 (3.14)	72 (1.87)	
Education levels, n (%)						*<0.001*
< high school	1303 (8.47)	247 (6.42)	289 (7.51)	333 (8.64)	434 (11.29)	
= high school	4594 (29.85)	1042 (27.14)	1239 (32.15)	1278 (33.21)	1035 (26.93)	
> high school	9491 (61.68)	2552 (66.44)	2326 (60.35)	2238 (58.14)	2375 (61.78)	
PIR, n (%)						*<0.001*
< 1	1976 (12.84)	454 (11.81)	507 (13.15)	492 (12.79)	523 (13.61)	
1-3	5038 (32.74)	1207 (31.43)	1262 (32.76)	1304 (33.87)	1265 (32.91)	
> 3	8374 (54.42)	2180 (56.76)	2085 (54.09)	2053 (53.34)	2056 (53.49)	
Smoking status, n (%)						
Never	7752 (50.38)	2310 (60.14)	2079 (53.94)	1919 (49.85)	1714 (44.59)	*< 0.001*
Former	3674 (23.88)	825 (21.47)	890 (23.09)	1007 (26.16)	952 (24.77)	
Current	3962 (25.75)	706 (18.39)	885 (22.97)	923 (23.99)	1178 (30.64)	
Alcohol consumption, n (%)						*<0.001*
Never	1733 (11.26)	494 (12.87)	440 (10.13)	358 (8.51)	441 (11.47)	
Former	2285 (14.85)	372 (9.68)	503 (13.04)	591 (15.35)	819 (21.31)	
Mild	6027 (39.17)	1478 (38.47)	1396 (36.21)	1572 (40.84)	1581 (41.13)	
Moderate	2341 (15.21)	830 (21.61)	664 (17.23)	566 (14.71)	281 (7.31)	
Heavy	3002 (19.51)	667 (17.37)	851 (22.11)	762 (19.80)	722 (18.78)	
BMI (kg/m²)	26.84 (23.45, 32.00)	23.85 (21.85, 28.34)	26.15 (22.95,30.84)	29.10 (26.60, 33.40)	29.30 (25.40, 34.56)	*< 0.001*
DM, n (%)						*<0.001*
No	10809 (70.24)	3275 (85.27)	2909 (75.49)	2547 (66.17)	2078 (54.06)	
IFG	1328 (8.63)	188 (4.89)	288 (7.47)	390 (10.14)	462 (1202)	
IGT	1470 (9.55)	181 (4.72)	326 (8.46)	373 (9.68)	590 (15.35)	
Yes	1781 (11.57)	197 (5.12)	331 (8.58)	539 (14.01)	714 (18.57)	
Hypertension, n (%)						*<0.001*
No	9364 (60.85)	3054 (79.50)	2562 (66.47)	2138 (55.54)	1610 (41.88)	
Yes	6024 (39.15)	787 (20.50)	1292 (33.53)	1711 (44.46)	2234 (58.12)	
Cancer, n (%)						*<0.001*
No	13640 (88.64)	3621 (94.27)	3451 (89.54)	3416 (88.75)	3152 (81.90)	
Yes	1748 (11.36)	220 (5.73)	403 (10.46)	433 (11.25)	692 (18.00)	
Hyperlipidemia, n (%)						*<0.001*
No	4850 (31.52)	2102 (54.72)	1266 (32.84)	587 (15.26)	895 (23.28)	
Yes	10538 (68.48)	1739 (45.28)	2588 (67.16)	3262 (84.74)	2949 (76.72)	
Lipiddrug, n (%)						*<0.001*
No	12521 (81.37)	3501 (91.14)	3249 (84.30)	3081 (80.04)	2690 (69.98)	
Yes	2867 (18.63)	340 (8.86)	605 (15.70)	768 (19.96)	1154 (30.02)	
Laboratory data						
FBG (mmol/L)	5.40 (5.07,5.85)	5.18 (4.67,5.45)	5.40 (5.05,5.78)	5.59 (5.20,5.94)	5.84 (5.30,6.85)	*< 0.001*
TG (mmol/L)	1.10 (0.85,1.70)	0.64 (0.52,0.75) 0.62 (0.50,0.72)	1.03 (0.91,1.13) 0.98 (0.85,1.10)	1.45 (1.30,1.63) 1.36 (1.20,1.58)	2.26 (1.93,2.80) 2.18 (1.86,2.70)	*< 0.001*
LDL-C (mmol/L)	2.85 (2.30,3.45)	2.51 (2.05,3.05)	2.89 (2.38,3.47)	3.00 (2.47,3.64)	3.05 (2.37,3.65)	*< 0.001*
ALT (U/L)	20.00 (15.00,27.00)	18.00 (15.00,2300)	20.00 (16.00,27.00)	23.00 (18.00,29.00)	25.00 (19.00,33.00)	*< 0.001*
AST (U/L)	22.00 (18.00,27.00)	21.00 (18.00,25.00)	22.00 (19.00,26.00)	23.00 (20.00,27.00)	23.00 (19.00,28.00)	*< 0.001*
WBC (10^9^/L)	6.20 (5.10,7.60)	5.70 (4.60,6.90)	6.10 (5.10,7.40)	6.40 (5.20,7.60)	6.90 (6.00,8.10)	*< 0.001*
Neutrophils (10^9^/L)	3.40 (2.70,4.40)	3.00 (2.40,3.90)	3.60 (3.30,4.50)	3.80 (3.10,4.70)	4.20 (3.20,5.00)	*< 0.001*
Monocytes (10^9^/L)	0.50 (0.40,0.60)	0.50 (0.40,0.60)	0.50 (0.40,0.60)	0.50 (0.40,0.60)	0.50 (0.40,0.80)	*< 0.001*
Lymphocytes (10^9^/L)	1.80 (1.50,2.20)	1.80 (1.50,2.20)	1.90 (1.50,2.20)	2.10 (1.70,2.50)	2.00 (1.70,2.60)	*< 0.001*
PLT (10^9^/L)	240.00 (200.00,285.00)	230.00 (200.00,268.00)	240.00 (205.00,284.00)	245.00 (208.00,288.00)	244.00 (202.00,289.00)	*< 0.001*

BMI, body mass index; PIR, poverty income ratio; IFG,impaired fasting glucose; IGT, impaired glucose tolerance; FBG, fasting blood glucose; TG, triglyceride; LDL-C, low-density lipoprotein cholesterol; ALT, alanine aminotransferase; AST, aspartate aminotransferase; WBC, white blood cell; PLT, platelet; TyG, triglyceride-glucose; NLR, neutrophil-lymphocyte ratio.

**Table 3:** Baseline characteristics of the study population based on TyG-NLR quartile grouping.

Variables	Total (N=15388)	Q1	Q2	Q3	Q4	*P*-value
Age group, n (%)	15388	3847	3846	3847	3848	*< 0.001*
< 60	10856 (70.55)	3125 (81.24)	3021 (78.56)	2801 (72.80)	1909 (49.61)	
≥ 60	4532 (29.45)	722 (19.56)	825 (21.44)	1046 (27.20)	1939 (50.39)	
Sex, n (%)						*<0.001*
Female	8076 (52.48)	2082 (54.13)	1881 (48.90)	2023 (52.58)	1954 (50.77)	
Male	7312 (47.52)	1765 (45.87)	1965 (51.10)	1824 (47.42)	1894 (49.23)	
Race, n (%)						*<0.001*
Non-Hispanic Black	1971 (12.81)	598 (15.54)	528 (13.74)	435 (11.31)	410 (10.65)	
Mexican American	1514 (9.84)	554 (14.41)	451 (11.72)	364 (9.45)	145 (3.77)	
Non-Hispanic White	11084 (72.03)	2342 (60.87)	2650 (68.91)	2881 (74.89)	3211 (83.45)	
Other Race	819 (5.32)	353 (9.18)	217 (5.64)	167 (8.35)	82 (2.13)	
Education levels, n (%)						*<0.001*
< high school	1303 (8.47)	224 (5.81)	267 (6.94)	318 (8.27)	494 (12.84)	
= high school	4593 (29.85)	976 (25.38)	1101 (28.63)	1305 (33.92)	1211 (31.47)	
> high school	9491 (61.68)	2647 (68.81)	3247 (84.43)	2224 (57.81)	1373 (35.68)	
PIR, n (%)						*<0.001*
< 1	1976 (12.84)	549 (14.26)	502 (13.05)	432 (11.24)	493 (12.81)	
1-3	5038 (32.74)	1481 (38.51)	1279 (33.25)	1140 (29.63)	1138 (29.57)	
> 3	8374 (54.42)	1817 (47.23)	2065 (53.70)	2275 (59.14)	2217 (57.61)	
Smoking status, n (%)						
Never	7752 (50.38)	2315 (60.17)	2118 (55.08)	1876 (48.77)	1443 (37.50)	*< 0.001*
Former	3674 (23.88)	817 (21.25)	907 (23.57)	1021 (26.54)	929 (24.14)	
Current	3962 (25.75)	715 (18.58)	821 (21.35)	950 (24.69)	1476 (38.36)	
Alcohol consumption, n (%)						*<0.001*
Never	1733 (11.26)	467 (12.14)	424 (11.02)	389 (10.11)	453 (11.77)	
Former	2285 (14.85)	417 (10.83)	482 (12.54)	601 (15.62)	785 (20.40)	
Mild	6027 (39.17)	1356 (35.24)	1488 (38.69)	1560 (40.55)	1623 (42.18)	
Moderate	2341 (15.21)	814 (21.18)	665 (17.28)	551 (14.32)	311 (8.08)	
Heavy	3002 (19.51)	793 (20.61)	787 (20.47)	746 (19.39)	676 (17.57)	
BMI (kg/m²)	26.84 (23.45,32.00)	25.82 (22.70,30.50)	26.50 (23.10,31.40)	28.85 (25.86,33.60)	28.87 (25.16,34.12)	*< 0.001*
DM, n (%)						*<0.001*
No	10809 (70.24)	2802 (72.83)	2717 (70.65)	2644 (68.74)	26469 (68.76)	
IFG	1328 (8.63)	255 (6.63)	309 (8.04)	383 (9.95)	381 (9.90)	
IGT	1470 (9.55)	265 (6.88)	332 (8.62)	407 (10.58)	466 (12.11)	
Yes	1781 (11.57)	525 (13.66)	488 (12.69)	413 (10.73)	355 (9.23)	
Hypertension, n (%)						*<0.001*
No	9364 (60.85)	2760 (71.74)	2635 (68.52)	2402 (62.43)	1567 (40.72)	
Yes	6024 (39.15)	1087 (28.26)	1211 (31.48)	1445 (37.57)	2281 (59.28)	
Cancer, n (%)						*<0.001*
No	13640 (88.64)	3572 (92.85)	3534 (91.89)	3439 (89.40)	3095 (80.43)	
Yes	1748 (11.36)	275 (7.15)	312 (8.11)	408 (10.60)	753 (19.57)	
Hyperlipidemia, n (%)						*<0.001*
No	4850 (31.52)	1435 (37.30)	1258 (32.70)	1011 (26.27)	1146 (29.78)	
Yes	10538 (68.48)	2412 (62.70)	2588 (67.30)	2798 (72.73)	2702 (70.22)	
Lipiddrug, n (%)						*<0.001*
No	12521 (81.37)	3440 (89.43)	3198 (83.14)	3110 (80.85)	2773 (72.06)	
Yes	2867 (18.63)	407 (10.57)	648 (16.86)	737 (19.45)	1075 (27.94)	
Laboratory data						
FBG (mmol/L)	5.40 (5.07,5.85)	5.35 (4.97,5.67)	5.40 (5.05,5.76)	5.53 (5.14,6.03)	5.64 (5.20,6.27)	*< 0.001*
TG (mmol/L)	1.10 (0.85,1.70)	0.91 (0.64,1.38)	1.08 (0.75,1.54)	1.26 (0.91,1.78)	1.29 (0.86,1.83)	*< 0.001*
LDL-C (mmol/L)	2.85 (2.30,3.45)	2.78 (2.28,3.41)	2.90 (2.32,3.50)	2.93 (2.27,3.52)	2.80 (2.21,3.34)	*< 0.001*
ALT (U/L)	20.00 (15.00,27.00)	20.00 (16.00,27.00)	20.00 (16.00,28.00)	21.00 (17.00,28.00)	20.00 (16.00,27.00)	*< 0.001*
AST (U/L)	22.00 (18.00,27.00)	22.00 (18.00,27.00)	22.00 (18.00,26.00)	22.00 (19.00,27.00)	22.00 (19.00,26.00)	*< 0.001*
WBC (10^9^/L)	6.20 (5.10,7.60)	5.40 (4.20,6.40)	5.90 (5.00,7.10)	6.50 (5.30,7.60)	7.60 (6.30,8.80)	*< 0.001*
Neutrophils (10^9^/L)	3.40 (2.70,4.40)	2.50 (2.10,3.00)	3.20 (2.70,3.90)	4.10 (3.60,4.80)	5.00 (4.20,6.00)	*< 0.001*
Monocytes (10^9^/L)	0.50 (0.40,0.60)	0.50 (0.40,0.60)	0.50 (0.30,0.60)	0.50 (0.30,0.60)	0.60 (0.40,0.70)	*< 0.001*
Lymphocytes (10^9^/L)	1.80 (1.50,2.20)	2.20 (1.80,2.60)	1.90 (1.70,2.30)	1.70 (1.60,2.10)	1.50 (1.30,1.80)	*< 0.001*
PLT (10^9^/L)	240.00 (200.00,285.00)	235.00 (200.00,270.00)	238.00 (206.00,283.00)	243.00 (204.00,285.00)	244.00 (201.00,286.00)	*< 0.001*

BMI, body mass index; PIR, poverty income ratio; IFG,impaired fasting glucose; IGT, impaired glucose tolerance; FBG, fasting blood glucose; TG, triglyceride; LDL-C, low-density lipoprotein cholesterol; ALT, alanine aminotransferase; AST, aspartate aminotransferase; WBC, white blood cell; PLT, platelet; TyG, triglyceride-glucose; NLR, neutrophil-lymphocyte ratio.

**Table 4:** Association of TyG and TyG-NLR indicators with all-cause mortality.

	Q1	Q2	Q3	Q4	*P for trend*
TyG					
Model 1	Ref	1.93(1.47–3.09)	2.08(1.37–2.85)	3.73(2.64–5.31)	*< 0.001*
Model 2	Ref	1.15(0.84–1.71)	0.92(0.64–1.22)	1.31(1.03–2.15)	*0.057*
Model 3	Ref	1.08(0.65–1.53)	0.79(0.51–1.15)	1.02(0.62–1.48)	*0.728*
TyG-NLR					
Model 1	Ref	0.75(0.46-1.18)	1.71(1.08-2.61)	3.27(2.11-4.27)	*< 0.001*
Model 2	Ref	0.71(0.41-1.10)	1.25(0.79-1.84)	1.82(1.14-2.57)	*< 0.001*
Model 3	Ref	1.05(1.01-1.24)	1.19(1.05-1.84)	1.63(1.08-2.54)	*< 0.001*

Model 1: crude model; Model 2: Adjusted for sex and age; Model 3: Adjusted for sex, age, race, PIR, educational levels, BMI, smoking status, alcohol consumption, hypertension, diabetes mellitus, hyperlipidemia, cancers, lipid-lowering drugs, ALT, and AST; TyG: triglyceride-glucose; NLR: neutrophil-lymphocyte ratio; CI: confidence interval; Ref: reference; PIR: poverty income ratio; BMI: body mass index;ALT: alanine aminotransferase; AST: aspartate aminotransferase.

### The dose-response relationships between the TyG and TyG-NLR indices and mortality were analyzed

**Figure 3:**
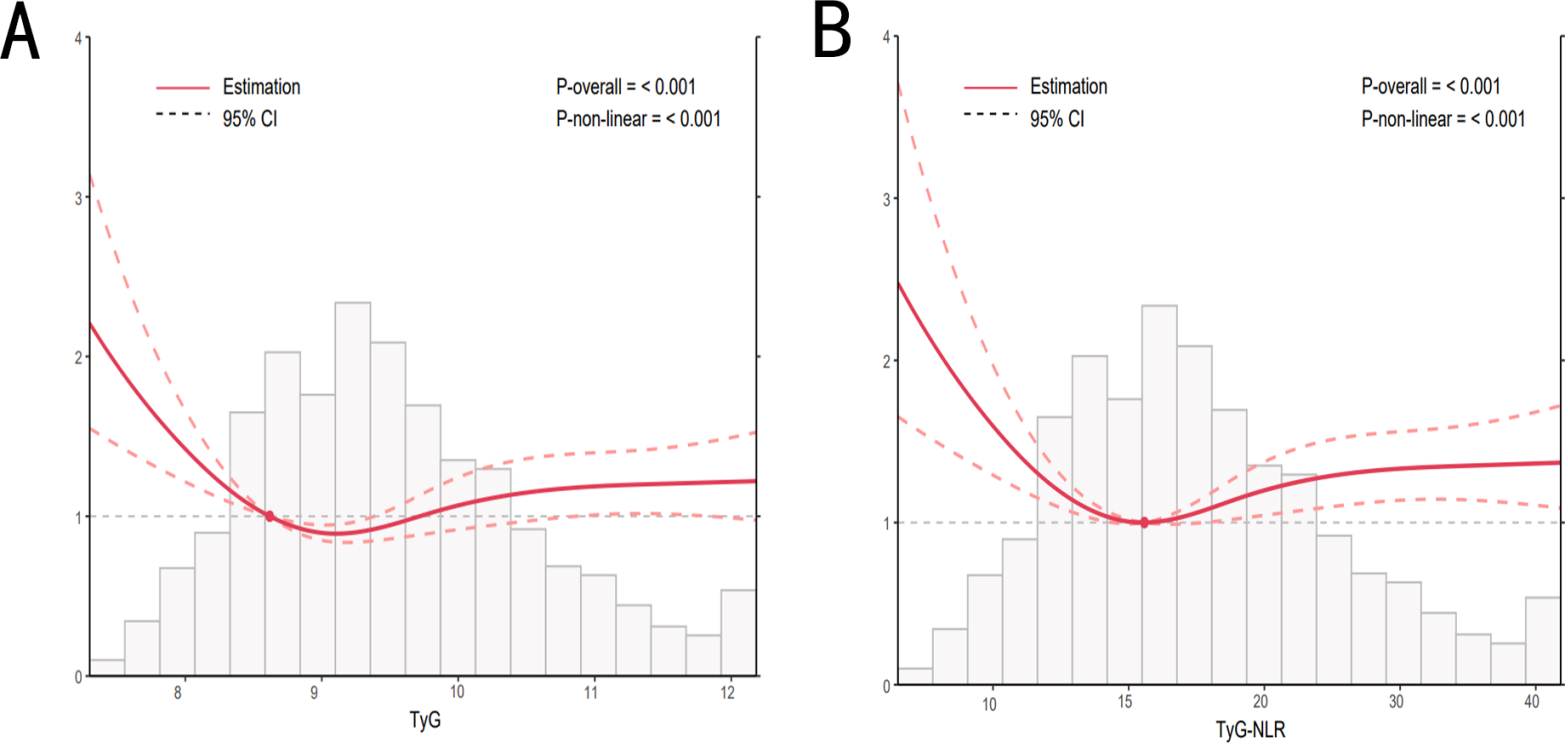
Dose-response relationship between TyG, TyG-NLR, and all-cause mortality. Adjusted for sex, age, race, PIR, educational levels, BMI, smoking status, alcohol consumption, hypertension, diabetes mellitus, hyperlipidemia, cancers, lipid-lowering drugs, ALT, and AST. TyG, triglyceride-glucose; NLR, neutrophil-lymphocyte ratio; PIR, poverty income ratio; BMI, body mass index; ALT, alanine aminotransferase; AST, aspartate aminotransferase.

After adjusting for potential confounders, we analyzed the dose-response relationship between the TyG and TyG-NLR indices and mortality using RCS analysis (consistent with Model 3). The dose-response relationships between both indices and all-cause mortality were nonlinear (*P* for nonlinearity < 0.05), as shown in **Figure 3**. When assessing the dose-response relationships between the TyG and TyG-NLR indices and mortality (**Figure 3**), a similar pattern emerged. Notably, regardless of the specific shape of the dose-response curve, once the respective threshold points were exceeded, an increase in both the TyG and TyG-NLR indices was associated with a higher risk of mortality.

## Discussion

IR and inflammation are closely associated with various metabolic diseases and adverse outcomes (24, 25). NLR reflects the systemic inflammatory status of the body and has been shown to be related to poor outcomes and prognosis in several studies (26, 27). Therefore, in this study, we combined the TyG index with NLR, an indicator of the degree of inflammatory response, to construct a new index, TyG-NLR, which may better assess the risk of mortality in the general population. Since both NLR and the TyG index are composite indicators derived from routine blood parameters, they can be easily obtained in clinical practice, making them highly feasible for practical clinical guidance.

Several previous studies have explored and analyzed the relationship between the TyG index and mortality rates in the general population, but there are inconsistencies in their findings (28–30). Yang et al. analyzed NHANES data from 2009 to 2018 and found that for each 1-unit increase in the TyG index, the risk of all-cause mortality increased by 25%. They also noted that the relationship between the TyG index and mortality risk was influenced by gender. However, no significant association was observed when the TyG index was analyzed as a categorical variable (31). Li et al. identified a threshold effect between the TyG index and mortality, finding that participants with higher TyG levels had a 1.67-fold increased risk of all-cause mortality compared to those with lower levels. However, when the TyG index was grouped into tertiles, this association disappeared (32). Although these studies identified a correlation between the TyG index and mortality rates, the results may have been influenced by factors such as small sample sizes and confounding variables. These findings suggest that the relationship between the TyG index and all-cause mortality may be inconsistent when used as a single predictor.

In our study, the TyG index showed an association with all-cause mortality before adjusting for confounders. However, this association became non-significant after adjusting for confounders, which is consistent with previous studies, demonstrating the instability of the TyG index when used alone (33). However, when we utilized the combined TyG-NLR index, our results showed that TyG-NLR was significantly associated with all-cause mortality. After adjusting for all confounders, participants in the highest quartile of the TyG-NLR index had a 1.63-fold increased risk of all-cause mortality compared to those in the lowest quartile. Additionally, we observed that participants with TyG-NLR indices in the second and third quartiles had 1.05-fold and 1.19-fold increased mortality risks, respectively, compared to those in the lowest quartile. These characteristics not only establish the TyG-NLR index as an effective tool for evaluating the risk of all-cause mortality but also further support the central role of insulin resistance and inflammation in adverse health outcomes, providing a crucial theoretical basis for future risk stratification, prediction, and intervention strategies.

We believe the positive correlation between the TyG-NLR index and mortality risk is driven by the following factors. First, chronic insulin resistance is not only highly correlated with systemic inflammation but also leads to lipid metabolism disorders, thereby accelerating the progression of atherosclerosis, which is considered the primary pathological basis of various cardiovascular diseases. For instance, insulin resistance can lead to endothelial dysfunction through multiple pathways, impairing normal vascular regulation and increasing the risk of cardiovascular events (34, 35). Second, NLR, as a sensitive indicator of systemic inflammatory status, can assess the body’s immune response capacity. Chronic inflammation may suppress normal immune function, making individuals more susceptible to infections or malignant diseases, which may directly or indirectly increase the risk of mortality. Additionally, the interaction between insulin resistance and inflammation may exacerbate tissue and organ damage and functional decline through elevated pro-inflammatory cytokines (such as TNF-α and IL-6) and oxidative stress levels, playing a critical role in the progression of various chronic diseases (36, 37).

Notably, our findings and conclusions require further validation in larger cohorts and diverse populations to determine whether they are influenced by participants’ socioeconomic environments, similar to the TyG index. Additionally, the biological mechanisms underlying the association between the TyG-NLR index and mortality warrant further investigation. In summary, both the TyG index and systemic inflammation markers can be measured through routine blood biochemical tests that are affordable and do not require complex equipment. Once the effectiveness and reliability of the TyG-NLR index are confirmed in future studies for assessing mortality risk in the general population, it could be considered for use in primary care and health screening. Risk stratification using the TyG-NLR index may help guide targeted therapeutic strategies to improve patient outcomes.

### Strengths and limitations

The strengths of this study lie in it being the first to propose the TyG-NLR index and confirm its association with mortality in the general population. Additionally, unlike previous studies that primarily focused on populations with metabolic disorders, our study demonstrates that the TyG-NLR index can serve as a predictor of mortality risk in the general population. However, this study has several limitations. First, as an observational cohort study, we cannot establish a direct causal relationship between the TyG-NLR index and mortality. Furthermore, the underlying mechanisms driving the significant association between the TyG-NLR index and mortality remain unclear. Lastly, since the study participants were from the United States, further validation is needed across different populations and regions.

## Conclusion

Our results indicate a significant positive association between the TyG-NLR index and all-cause mortality. This simple, accessible, and cost-effective index holds potential as a marker for identifying mortality risk in the general population in clinical practice.

## Data Availability

The original contributions presented in the study are included in the article/supplementary material. Further inquiries can be directed to the corresponding author.
